# Extracellular Vesicles and Asthma—More Than Just a Co-Existence

**DOI:** 10.3390/ijms22094984

**Published:** 2021-05-07

**Authors:** Bilal Alashkar Alhamwe, Daniel P. Potaczek, Sarah Miethe, Fahd Alhamdan, Lukas Hintz, Arslan Magomedov, Holger Garn

**Affiliations:** 1 Institute of Tumor Immunology, Clinic for Hematology, Oncology and Immunology, Center for Tumor Biology and Immunology, Philipps University Marburg, 35043 Marburg, Germany; bilal.alashkaralhamwe@staff.uni-marburg.de (B.A.A.); arslan.magomedov@med.uni-giessen.de (A.M.); 2 College of Pharmacy, International University for Science and Technology (IUST), Daraa 15, Syria; 3 Translational Inflammation Research Division & Core Facility for Single Cell Multiomics, Member of the German Center for Lung Research (DZL) and the Universities of Giessen and Marburg Lung Center, Philipps University Marburg, 35043 Marburg, Germany; danppot@gmail.com (D.P.P.); sarah.miethe@staff.uni-marburg.de (S.M.); alhamdan@students.uni-marburg.de (F.A.); hintz@staff.uni-marburg.de (L.H.)

**Keywords:** airway, allergy, asthma, epigenetic(-s), exosome, extracellular vesicle (EV), inflammation, microRNA (miRNA), microvesicle (MV)

## Abstract

Extracellular vesicles (EVs) are membranous structures, which are secreted by almost every cell type analyzed so far. In addition to their importance for cell-cell communication under physiological conditions, EVs are also released during pathogenesis and mechanistically contribute to this process. Here we summarize their functional relevance in asthma, one of the most common chronic non-communicable diseases. Asthma is a complex persistent inflammatory disorder of the airways characterized by reversible airflow obstruction and, from a long-term perspective, airway remodeling. Overall, mechanistic studies summarized here indicate the importance of different subtypes of EVs and their variable cargoes in the functioning of the pathways underlying asthma, and show some interesting potential for the development of future therapeutic interventions. Association studies in turn demonstrate a good diagnostic potential of EVs in asthma.

## 1. Introduction

Chronic non-communicable diseases (NCDs) are inflammatory conditions, which are not caused by infectious agents (e.g., bacteria, viruses, parasites). To name a few, these diseases include respiratory disorders such as asthma or chronic obstructive pulmonary disease (COPD), chronic inflammatory bowel diseases, cardiovascular disorders such as coronary artery disease/ischemic heart disease, peripheral vascular disease or stroke, all based on atherosclerosis, inflammatory disease conditions in the skin (e.g., atopic dermatitis, psoriasis), metabolic diseases such as obesity, metabolic syndrome, and diabetes, different forms of cancer, adverse mental outcomes, etc. Especially after the development of effective prevention (vaccines) and treatment (e.g., antibiotics) options against infectious diseases over the last decades, NCDs became the most significant cause of death in the world. According to the World Health Organization (WHO), in 2019 the three top causes of death in the World were ischemic heart disease accounting for about 9 million, stroke for more than 6 million deaths, and COPD for more than 3 million deaths in this single year only [1]. For comparison, as of mid-April 2021, the worldwide number of COVID-19-related deaths since the very beginning of the pandemic was approaching 3 million [2]. The burden of NCDs is high in western countries and still rising, in particular in less developed areas [3,4,5,6]. To effectively face this challenge, novel diagnostic and therapeutic approaches should be established based on the growing knowledge on pathobiological mechanisms underlying the development and the clinical course of NCDs.

This specifically applies also to asthma as one of the most prominent NCDs, for which, despite substantial progress, current diagnostic and therapeutic approaches remain suboptimal. One of the major reasons behind this is the heterogeneity of asthma, with a complex etiology and multiple clinical representations, requiring the development of stratified diagnosis and treatment strategies [7,8,9,10]. These can only be achieved on the basis of novel cellular and molecular insights based on innovative methods. In this review, we summarize the current knowledge on extracellular vesicle (EV)-mediated cell-cell communication obtained in the context of pathobiology and clinical pathology of asthma.

## 2. Asthma

Asthma is a chronic inflammatory disease of the airways characterized by recurrent symptoms of varying intensity and severity, including wheezing, shortness of breath, cough, feeling of tightness in the chest, and others. The symptoms of asthma are underlain by reversible airway obstruction resulting from easily triggered bronchospasm and enhanced mucus secretion. In a longer perspective, disease progression is associated with, or rather results from, airway remodeling including changes in structural cell composition and extracellular fibrosis [11,12,13,14].

Clinically, asthma is a very heterogeneous disorder with considerable differences in the symptomatology, factors triggering exacerbations, severity, time of onset, demographics, body weight, and other features. Characteristic clinical representations of asthma form so-called phenotypes that are associated with a variety of distinct pathomechanisms named endotypes. Several endotypes have been proposed, which can be roughly grouped into those related to T helper cell type-2 (Th2) and those related to non-Th2 (e.g., Th1/Th17) immune mechanisms. Since it became evident that Th cytokines can be secreted also by other cell types, e.g., innate lymphoid cells (ILCs), asthma forms are divided into those of a type-2 (mostly allergic) and those of a non-type-2 character, respectively. However, even this paradigm may not cover all possible mechanisms underlying different forms of asthma [8,9,13,15,16].

**Figure 1:**
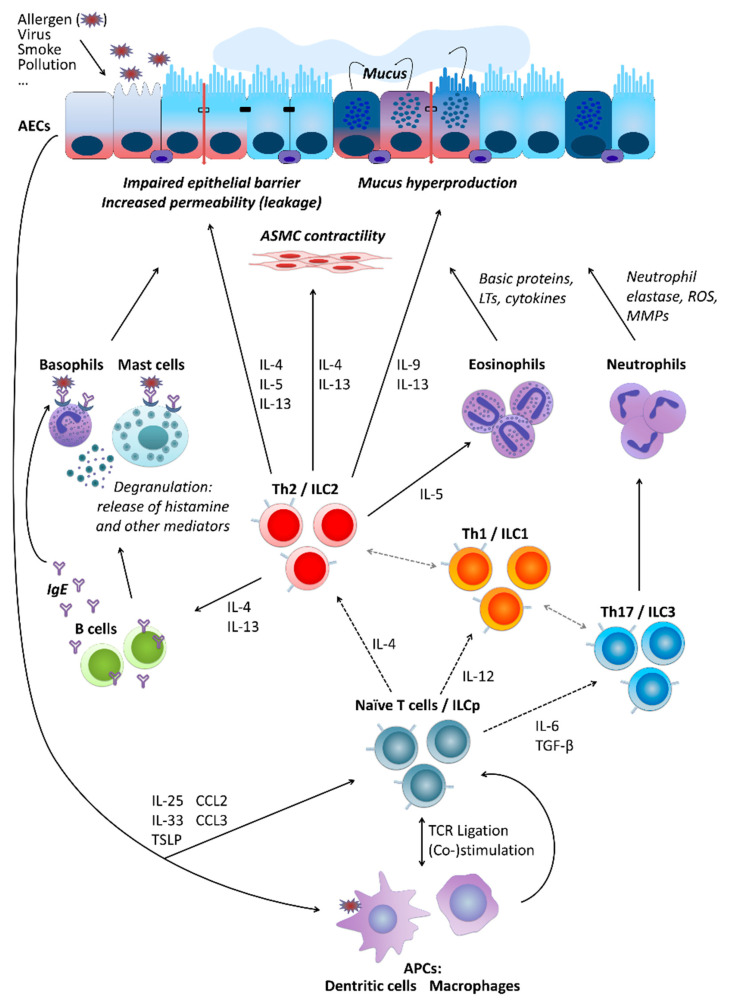
Basic cellular mechanisms underlying type-2 and non-type-2 asthma. For a more detailed description, please refer to the main text, Section 2. “Asthma”. AECs, airway epithelial cells; ASMC, airway smooth muscle cells; LTs, leukotrienes; ROS, reactive oxygen species; MMPs, matrix metalloproteinases; IL, interleukin; ILC, innate lymphoid cells; ILCp, ILC precursors; Th (cells), T helper (cells); IgE, immunoglobulin E; TGF-β, transforming growth factor beta; TSLP, thymic stromal lymphopoietin; CCL, C-C motif chemokine ligand; TCR, T cell receptor; APCs, antigen-presenting cells.

Partly independently of the pathomechanism behind it, several types of cells are crucially involved in asthma pathogenesis. These include airway epithelial cells (AECs) forming together with local macrophages the first point of contact for external influences entering the airways, for instance, allergens (type-2/atopic forms of asthma) or cigarette smoke (neutrophilic asthma belonging to non-type-2 disease forms). Cytokines secreted by AECs (e.g., thymic stromal lymphopoietin, TSLP; interleukin-25, IL-25; and IL-33) in response to stimulation influence of downstream cells including, among others, antigen-presenting cells (APCs) and T cells. Depending on the type of stimulation, T cells differentiate towards Th2 cells secreting cytokines driving allergic forms of the disease or Th17 and Th1 cells contributing to non-type-2 asthma endotypes. As type-2 cytokines, IL-4 triggers the differentiation of further Th2 cells and the production of immunoglobulin E (IgE) by B cells, IL-13 activates mast cells and basophils as well as stimulates airway smooth muscle (ASM) cell contractility and thus airway hyper-responsiveness (AHR) and hyperplasia of goblet cells and mucus production, IL-5 activates eosinophils, IL-9 further contributes to increased mucus production and enhanced proliferation of mast cells. Mediators secreted by mast cells and basophils result in allergic inflammation of the respiratory tract accompanied by a respective clinical picture. IL-17 released by Th17 cells, in turn, stimulates neutrophil activation, which leads to severe endothelial injury typical for non-type-2 neutrophilic asthma (Figure 1) [9,14,17,18,19].

## 3. Extracellular Vesicles

A key feature that has significantly contributed to the evolution of multicellular organisms and especially higher levels of complexity is represented by the ability of intercellular communication such as transfer of soluble molecules between cells and/or direct cell-cell contact. After the discovery that cells release so-called apoptotic bodies during programmed cell death, it was shown already in the mid-60s that physiologically active cells also release extracellular particles, at that time referred to as the so-called “platelet dust” [20]. However, within the last two decades, EVs have turned out to be more prominent and functionally important than initially expected and emerged as an interesting and promising research field. Virtually all cell types analyzed so far release EVs, which can roughly be classified into two major groups: endosomal derived exosomes and microvesicles (MVs; also referred to as microparticles or ectosomes), the latter directly budding from the plasma membrane [21,22,23,24,25]. Although exosomes and MVs show differences, such as their biogenesis and release pathways, they also share many bio-physicochemical properties, including size range, density as well as certain surface proteins (for a summary of the differential characteristics of EVs see Table 1) [26,27,28,29,30,31,32,33]. These features barely allow distinguishing between the individual subpopulations in detail. Instead of referring to the individual subpopulations, the term EV should therefore be preferred in the nomenclature, which encompasses vesicles released by cells in their entirety [34], however, in the current review we will retain the terminology used in the original publications to which we refer.

Since EVs play an important role in cell-cell communication, they are not simply empty lipid bins but rather contain various biomolecules such as diverse RNA types, proteins, lipids and metabolites by which they have the potential to regulate the function of recipient cells. With respect to EV-mediated signaling, non-coding RNAs were studied in depth during the last decade. In particular, the role of microRNAs (miRNAs) turned into the focus of research, due to their well-established role in the regulation of gene expression [35,36,37]. Interestingly, the way in which EVs avoid degradation while entering the cell compartment by endocytosis and subsequent cargo release via membrane fusion suggests that EVs exploit mechanisms similar to those observed in certain viral infections, such as endosomal acidification [38,39].

In line with this, recent studies also implicated EVs in the progression of human disease, including cancer and infectious diseases (for a summary see [40,41]).

## 4. MicroRNA

Epigenetic mechanisms alter the expression of genes without affecting DNA nucleotide sequences. There are different types of epigenetic control, which can be roughly divided into classical and non-classical epigenetic mechanisms. Classical epigenetic mechanisms include DNA methylation and posttranslational histone modifications, such as acetylation, methylation, phosphorylation, and others [17,42]. Non-classical epigenetic mechanisms are mediated by post-transcriptional control elements including non-coding transcripts, such as miRNAs, approximately twenty-two nucleotides-long RNA molecules that can also be detected as cargoes of EVs [17,43].

**Figure 2:**
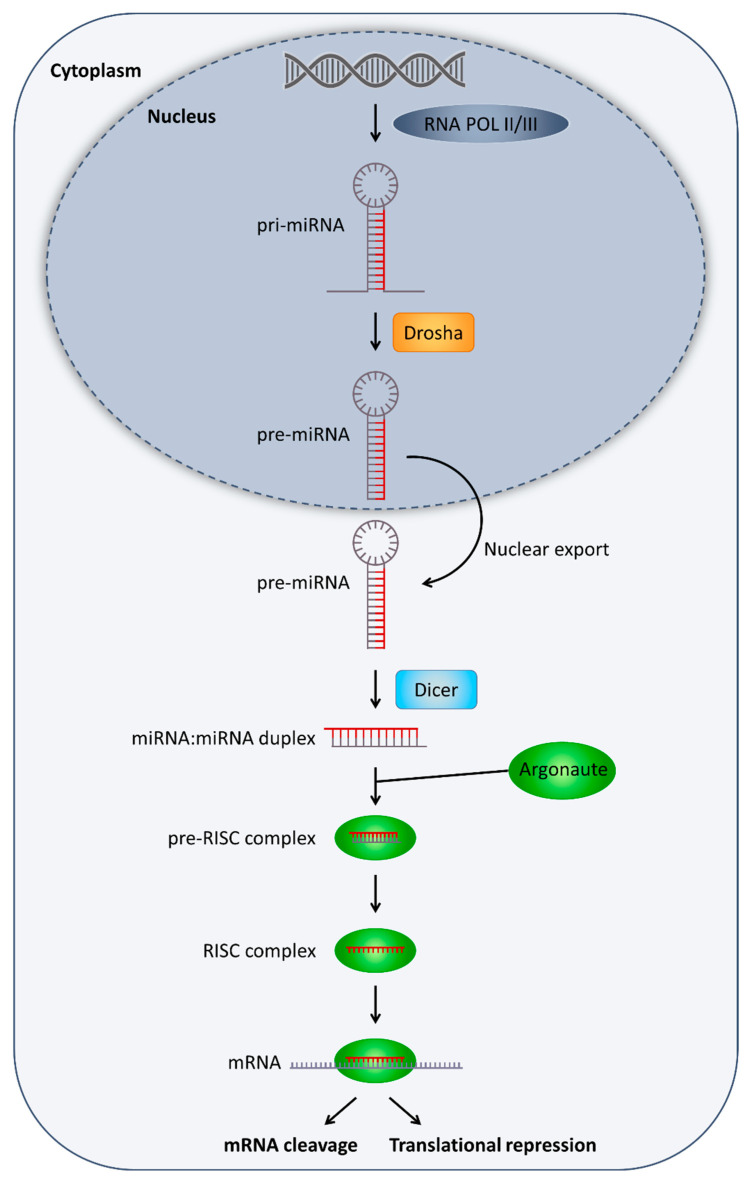
Basic processes involved in microRNA (miRNA) biogenesis and function. For detailed description, please refer to the main text, Section 4. “MicroRNA”. RISC, RNA-induced silencing complex; mRNA, messenger RNA.

With the advent of high-throughput sequencing technologies, the number of known miRNAs in the human genome has steadily increased during recent years, so that currently approximately 2500 miRNA sequences are known. The canonical miRNA molecules are located within non-coding introns, coding exons as well as intergenic regions. The transcription of miRNAs is commonly mediated by RNA Polymerase II (RNA Pol II), but in a few cases also by RNA Pol III. All miRNAs undergo a series of maturation steps, in which the immature transcripts are processed by type III RNases. More precisely, primary (pri)-miRNAs are processed by Drosha within the nucleus to pre-miRNAs and after export into the cytoplasm, further by Dicer into active mature approximately 22-nt long miRNAs (Figure 2) [44,45,46,47].

After processing, one strand of the mature miRNA is loaded into the RNA-induced silencing complex (RISC), where it ensures the specific recognition of a target mRNA. Gene expression is mainly regulated by binding of RISC to the 3′ untranslated region (UTR) of the mRNA, which can lead to translation inhibition or even degradation of the mRNA. The two mechanisms are mainly regulated by the complementarity of the miRNA to the target sequence. Thus, complete binding of the miRNA results in the fragmentation of the mRNA (Figure 2), which is induced by Argonaute 2 (Ago2) a component of the RISC complex [44,45,46,47].

Importantly, miRNAs are considered as one of the key regulators of literally all cellular pathways [17,45,48,49].

## 5. Extracellular Vesicles and Asthma: Cellular Level

### 5.1. Airway Epithelial Cells and Fibroblasts

EVs are involved in asthma-related interactions between different cell types. Additionally, for AECs, the exchange of EV cargo seems to be an important way of communicating with each other, as well as with other cell types. For example, in primary human tracheobronchial cells and cultured Calu-3 cells, a respiratory epithelial cell line, the reciprocal transfer of EV-associated proteins and miRNAs was shown to be sufficient to qualitatively and quantitatively alter the profiles of airway secretions including miRNA cargo of EVs of the target cells and cause mucin hypersecretion [50]. This mechanism may play an important role in epithelial remodeling and other pathologic processes in the airways involved in chronic inflammatory disorders of the respiratory tract, such as asthma, cystic fibrosis, and bronchogenic carcinoma [50]. In a mouse study, it was shown that the composition of the pool of extracellular miRNAs in the lung was very similar to that of the airway epithelium, with 80% of the EVs detected in bronchoalveolar lavage fluid (BALF) being of epithelial origin [51]. However, the number of miRNAs selectively expressed by immune cells, including miR-223 and miR-142a, and hematopoietic cell-derived EVs increased significantly following the induction of allergic airway inflammation (AAI), showing the importance of alterations in the EV miRNA pool for the development of allergic inflammation [51]. Another group reported that EV secretion and production of EV-associated proteins were both higher in the lungs of mice in which AAI was induced compared to the control animals [52]. These EVs, which were released during asthma/AAI by AECs under the influence of type-2 cytokines such as IL-13, triggered the proliferation and chemotaxis of undifferentiated macrophages [52]. Not surprisingly, the use of GW4869, an inhibitor of exosome production, resulted in a reduction in the population of proliferating monocytes in the AAI mouse model and the alleviation of various asthmatic features [52].

In another in vitro study, EV-associated miRNAs were released to the basal and apical sides by normal human bronchial epithelial cells (NHBECs) undergoing IL-13 treatment to mimic a type-2 inflammatory condition [53]. Candidate miRNAs identified were subsequently validated in EVs isolated from nasal lavages obtained from children with non-severe or severe asthma compared to healthy controls. It was shown that miR-92b, miR-210, and miR-34a expression levels correlated with the changes in lung function [53]. Treatment with IL-13 was also able to increase the expression of tissue factor (TF) and a release of TF-positive EVs from NHBECs, both induced by compressive stress mimicking asthmatic bronchoconstriction [54,55]. Besides, allergen challenge increased the release of TF into BALF in both mice and humans [54]. Although TF is best known for its role as a cellular initiator of coagulation [56,57], this multifunctional protein has also been implicated in a number of pathophysiologic conditions such as asthma [58,59,60].

**Figure 3:**
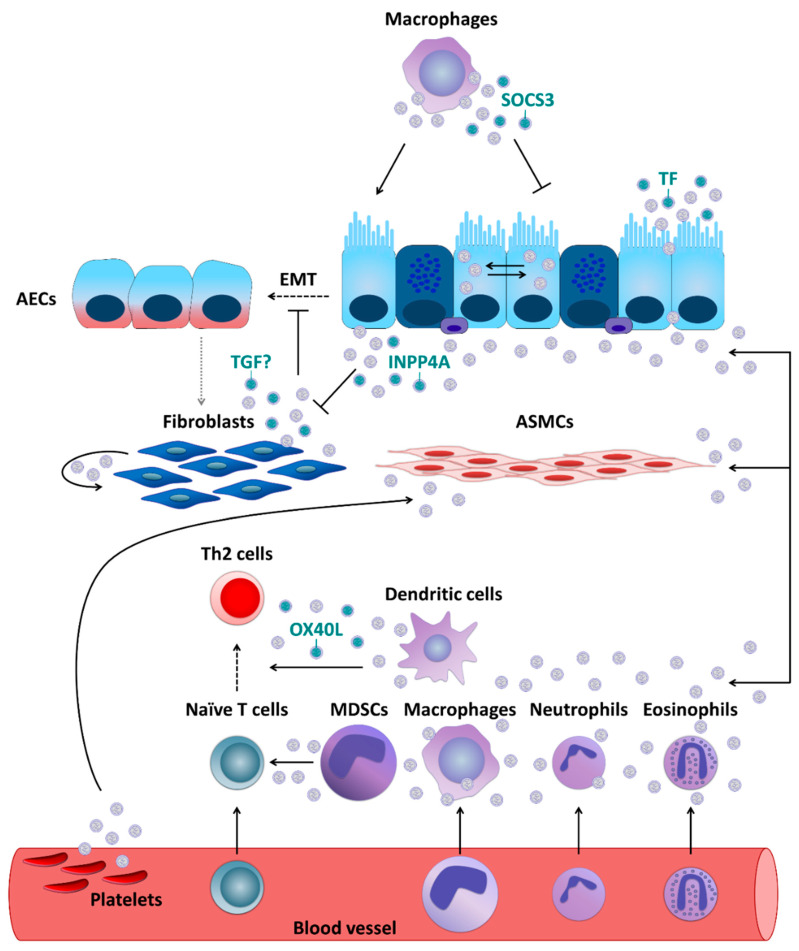
Extracellular vesicle- (EV-) mediated communication between cells crucial for asthma pathobiology. If not otherwise stated, EVs are thought to carry their usual content such as microRNAs, proteins, lipids, etc. For a more detailed description, please refer to the main text, Section 5.1 “Airway Epithelial Cells and Fibroblasts”, Section 5.2 “Antigen-Presenting Cells”, Section 5.3 “Granulocytes and Mast Cells”, and Section 5.4 “Platelets”. SOCS3, suppressor of cytokine signaling 3; TF, tissue factor; EMT, epithelial-mesenchymal transition; INPP4A, inositol polyphosphate 4-phosphatase type I A; OX40L, OX40 ligand; MDSCs, myeloid-derived suppressor cells; otherwise, please, refer to the legend to Figure 1.

Additionally, primary human fibroblasts were demonstrated to secrete exosomes, which undergo subsequent internalization by NHBECs [61]. Moreover, compared to healthy controls, exosomes derived from fibroblasts which were obtained from severe asthmatics showed lower levels of transforming growth factor beta 2 (TGF-β2) and significantly increased the proliferation of NHBECs [61]. These results are intriguing, given that TGF-β is considered to be a major driver of abnormal epithelial-mesenchymal transition (EMT) [9,62]. During EMT, epithelial cells demonstrate enhanced motility and invasive capacity through the downregulation of epithelial markers and higher expression of mesenchymal proteins, being this way a source of migrating myofibroblasts and fibroblasts. In turn, these cells promote extracellular matrix deposition and subepithelial fibrosis, which strongly contributes to the establishment of a persistent asthma phenotype [63,64]. Moreover, fibroblasts themselves can also be recipients of EVs. In vitro experiments using cell lines demonstrated that AECs were able to secrete enzymatically active inositol polyphosphate 4-phosphatase type I A (INPP4A) in EVs and as a soluble free form. INPP4A was then transferred to lung fibroblasts, and inhibition of such transfer resulted in increased fibroblast proliferation [65]. Moreover, in mice with or without AAI neutralization of extracellular INPP4A-induced AHR, with prominent airway remodeling, subepithelial fibroblast proliferation, and collagen deposition [65]. EV-mediated interactions between major cellular players involved in asthma/AAI (reported in Section 5.1, Section 5.2, Section 5.3, Section 5.4 and Section 5.5) are summarized in Figure 3.

### 5.2. Antigen-Presenting Cells

APCs, such as dendritic cells (DCs), macrophages, monocytes, and others can communicate through EVs with other types of cells involved in asthma development. A study performed in primary human macrophages and DCs demonstrated that they can secrete exosomes which contain enzymes for leukotriene biosynthesis and thus contribute to chronic inflammation, for example through granulocyte recruitment [66]. Primary human DCs activated with TSLP, an epithelial cell-derived cytokine [67], release exosomes expressing OX40 ligand (OX40L), which was able to promote proliferation and differentiation of CD4^+^ T cells towards a Th2 phenotype [68]. Resident alveolar macrophages were, in turn, demonstrated to dampen inflammatory signaling in AECs [69] and thus AAI in a mouse model [70] through transcellular delivery of suppressor of cytokine signaling 3 (SOCS3) within EVs. Air pollutants such as particulate matter are well-known contributors to the pathogenesis of chronic inflammatory airway diseases including asthma [17,71]. In vitro exposure to particulate matter stimulated human macrophages to release more EVs in a dose-dependent manner. Moreover, those EVs were able to induce secretion of pro-inflammatory cytokines, such as IL-6 and tumor necrosis factor-α (TNF-α), by pulmonary epithelial cells [72].

### 5.3. Granulocytes and Mast Cells

Likewise, human eosinophils were found to be able to secrete exosomes, the production of which was higher by cells deriving from asthmatics [73]. In addition, exosomes secreted by the eosinophils of patients with asthma could, in an autocrine manner, modify several specific eosinophil functions related to asthma pathogenesis including an increase in reactive oxygen species and nitric oxide synthesis and an augmentation of eosinophil migration and adhesion, suggesting that they could fundamentally contribute to the development and maintenance of asthma [74]. Further, asthmatic eosinophil-derived exosomes could enhance the apoptosis of primary AECs and delay the repair of established epithelial damage, as well as increase the proliferation of primary bronchial smooth muscle cells and perpetuate airway inflammation status [75]. Upon in vitro stimulation with LPS, horse neutrophil-derived exosomes, carrying proteins associated with immune response and positive regulation of cell communication, were rapidly internalized by equine ASM cells and enhanced their proliferative capabilities [76]. The effects of neutrophil-derived exosomes on ASM proliferation [76] might play an important role in the neutrophil-mediated progression of asthma and promotion of airway remodeling in severe and corticosteroid-insensitive patients with asthma [8]. Based on in vitro data obtained using human cells, exosomes were also suggested to partially contribute to mast cell-mediated pro-inflammatory modulation of ASM cells, although it was undisputed that soluble, extra-exosomal factors such as IL-8 were critical for the effect [77].

### 5.4. Platelets

Moreover, platelets, which are known to contribute to the pathophysiology of asthma as well [78,79], can exert their effects through EVs [80]. Plasma EVs, a substantial portion of which is of platelet origin, isolated from asthmatics were found to be able to reduce the endothelium-dependent relaxation in response to bradykinin and increase the acetylcholine-induced contraction of the mouse trachea, which is suggestive of their potential role in ASM dysfunction typical for asthma [81,82]. Moreover, the levels of circulating platelet microparticles (PMPs) were reported to be increased in asthmatics [83].

### 5.5. Myeloid-Derived Suppressor Cells

Myeloid-derived suppressor cells (MDSCs) comprise a heterogeneous group of immature myeloid cells that are capable of regulating T cell function through various mechanisms. It is hypothesized that they are involved in the regulation of respiratory inflammation in asthma [84]. Interestingly, EVs isolated from primary MDSCs of asthmatics and non-asthmatics have been reported to contain mitochondria, with a higher mitochondrial content in EVs derived from asthmatics [85]. Beyond that, these EVs could be transferred ex vivo to T cells, where they co-localized with the cellular mitochondrial network [85]. Mitochondrial transfer via EVs from MDSCs to T cells could represent an important novel regulatory mechanism contributing to the pathophysiology of asthma [85].

### 5.6. Mesenchymal Stem Cells and Adipose Tissue

Mesenchymal stem cells (MSCs) are multipotent stromal cells that possess self-renewing capacity and give rise to many specialized cell types such as chondrocytes, adipocytes, myocytes, and osteoblasts. MSCs are primarily located within the bone marrow (BM) stroma, but they can be isolated from almost any organ [86,87,88]. MVs produced by horse amniotic MSCs reduced the secretion of TNF-α and, to a certain extent, TGF-β and IL-6 from primary equine alveolar macrophages [89]. Exosomes secreted by human BM-derived MSCs were, in turn, able to upregulate secretion of IL-10 and TGF-β1 from peripheral blood mononuclear cells (PBMCs) of asthmatic patients, thus promoting proliferation and immunosuppressive capacity of regulatory T cells (Tregs). This effect was most probably mediated by APCs, shown to directly interact with exosomes produced by MSCs [90]. Furthermore, EVs deriving from mouse and human BM-MSCs turned out to ameliorate the mixed Th2/Th17 phenotype in an *Aspergillus*-induced mouse model of AAI reflective of severe refractory asthma [91]. In another in vitro study, miR-1470-containing exosomes from human MSCs were found to be able to promote the differentiation of Tregs among CD4^+^ T cells separated from PBMCs of acute asthmatic patients [92].

Exosomes from mouse adipose tissue-derived MSCs could effectively suppress the maturation of BM-derived DCs as reflected by decreased IL-6 release but augmented IL-10 and TGF-β secretion. In addition, lymphocyte proliferation was diminished in the presence of DCs treated with MSC-derived exosomes [93]. Treatment with EVs secreted by human adipose tissue-derived MSCs reduced the symptoms as well as cellular and molecular features of ovalbumin (OVA)-induced AAI in mice; lung TGF-β levels were also reduced [94]. Another study demonstrated that the attenuating effect of exosomes secreted by mouse adipose tissue-derived MSCs on airway remodeling observed in a mouse model of OVA-induced AAI could be further augmented by genetic modifications of the cells affecting the composition of the secreted exosomes [95]. Finally, adipocytes were found to be able to secrete fatty acid-binding protein 4 (FABP4; also called aP2) in exosome-like vesicles [96]. FABP4 was found to be necessary for AAI development in an OVA-based mouse model [97], which might represent a further link connecting (fat) metabolism and asthma [8]. Table 2 summarizes asthma/AAI-related cellular effects of EVs released by MSCs.

## 6. Extracellular Vesicles and Asthma: Higher Levels of Organization

### 6.1. Lower Respiratory Tract

Substantial differences in BALF exosomal miRNA profiles were observed between healthy subjects and patients with unprovoked, mild, stable asthma. Those referred to twenty-four miRNAs including members of the let-7 and miRNA-200 families, with a subset of sixteen miRNAs driving the robust separation between healthy control subjects and asthmatic patients [98]. Another study reported altered small RNA cargo with a lower load of miRNA in BALF exosomes obtained from severe asthmatics compared to healthy controls. Moreover, altered miRNA content of exosomes from severe asthmatics was predicted to be involved in the regulation of cellular pathways participating in airway inflammation and remodeling and correlated with lung function and the magnitude of BAL eosinophil and neutrophil infiltrations [99]. In contrast, a study performed in a mouse model of house dust mite (HDM)-induced AAI demonstrated the amount of airway-secreted EVs to be much higher in BALF from HDM-exposed mice compared to that from control animals [100]. HDM stimulated the secretion of exosomes containing increased levels of miRNAs inhibiting key molecules of type-2 inflammation, including the IL-5 receptor and IL-13. While the expression of those miRNAs was reduced in lung tissues, miRNAs that were less abundant in the exosomes were overrepresented in the lungs [100]. Pretreatment with the above-mentioned exosome secretion inhibitor GW4869 was able to diminish the number of exosomes secreted into BALF. Furthermore, the application of GW4869 also decreased type-2 cytokines and eosinophil counts in BALFs and reduced eosinophil accumulation in airway walls and mucosa [100]. Taken together, the results of this study suggest a very interesting mechanism in which selective sorting of miRNA, predominantly miRNAs inhibiting type-2 molecules, into airway-secreted EVs and their increased release to the airways after HDM exposure, i.e., removing them from the cells, could be involved in the pathogenesis of AAI [100]. Interestingly, analysis of BALF exosomes in mice subjected to an OVA-based model of AAI treated with scorpion and centipede demonstrated that BALF exosomal miRNAs might be involved in mediating the anti-asthmatic effects of these traditional Chinese insect medicines [101].

Moreover, other components of asthmatic EVs were also studied. Exosomal proteomics was analyzed in BALF obtained from patients with asthma, cystic fibrosis, or primary ciliary dyskinesia [102]. Mass spectrometry analysis made it possible to quantify 665 proteins, 14 of which were significantly differentially expressed depending on the disease condition. Furthermore, hierarchical analysis proved those 14 proteins to nicely distinguish the three diseases, with cystic fibrosis and primary ciliary dyskinesia having more similar protein profiles than had any of the two conditions compared with asthma [102]. Another study contrasted the lipid composition and presence of specific lipid mediators in EVs obtained from the BALF of healthy controls and asthmatic subjects exposed or not exposed to second-hand smoke [103]. Concentrations of BALF exosomes were higher in asthmatics, where they correlated with serum IgE levels and blood eosinophil counts. Phosphatidylglycerol, ceramide-phosphates, and ceramides were significantly more abundant in exosomes obtained from asthmatics compared to the non-exposed control groups. On the other hand, levels of sphingomyelin 34:1 were lower in exosomes of asthmatics with second-hand smoke exposure compared to healthy controls [103]. BALF exosomes were also compared between patients with mild asthma with an allergy against birch pollen and healthy individuals with exosomes obtained from asthmatics, and were characterized by higher levels of exosome-associated surface markers such as the tetraspanins CD63 and CD81 compared to those isolated from healthy controls [104]. Allergen provocation exerted no major effects on the characteristics of the exosomes. BALF exosomes contained enzymes for leukotriene biosynthesis and, especially if obtained from asthmatics, were able to promote the secretion of leukotriene C4 and IL-8 from the human bronchial epithelial cell line 16HB14o [104].

Asthma-related EV content has also been studied in sputum. It was demonstrated that exosomes could be detected in induced sputum of mild allergic asthmatics both before and after the challenge with an allergen [105]. The presence of RNA, especially short RNA species, and immune-related proteins in the samples as well as easy accessibility of induced sputum compared with BALF suggested sputum EV to be potential candidates for biomarker research in asthma [105].

### 6.2. Upper Respiratory Tract

Nasal exosomes have also been investigated in the context of asthma. Exosomal proteins and their potential functional properties were analyzed in nasal lavage fluid (NLF) obtained from asthmatics, subjects with asthma accompanied by chronic rhinosinusitis (CRS), and healthy individuals [106]. Strong associations were observed between the exosomal proteome and immune-related functions of NFL such as immune cell trafficking. Furthermore, while barrier-related proteins and those with antimicrobial functions were less abundant, serum-associated proteins and mucins were found to be present at higher levels in the exosomes obtained from patients suffering from respiratory diseases compared to healthy individuals. Finally, nasal exosomes were able to in vitro induce migration of various immune cells, including monocytes, neutrophils, and natural killer (NK) cells [106]. In another study, MV types and levels were comparatively analyzed in NLF samples obtained from healthy individuals and subjects with CRS without nasal polyps, CRS with nasal polyps, and aspirin-exacerbated respiratory disease (AERD), which is a syndrome comprising CRS with nasal polyps, asthma, and aspirin sensitivity [107]. Analysis of the released MVs demonstrated that mast cells, platelets, and basophils were more activated in subjects with AERD compared to patients with CRS, whereas epithelial injury was lower in CRS subjects with nasal polyps than in those with CRS without nasal polyps or AERD. These findings show that typing of NLF MVs might help to identify phenotypes of CRS and in distinguishing AERD from CRS with nasal polyps [107]. It was also observed that, independently of the presence or absence of concomitant asthma, exosomes released by primary human nasal epithelial cells obtained from patients suffering from CRS with nasal polyposis carried proteins influencing cell proliferation pathways and potentially leading to remodeling of the sinonasal mucosa [108].

### 6.3. Blood

Several studies focused on serum EVs and their importance for asthma. Mice subjected to an OVA-based model of AAI were first pretreated with either serum or exosomes isolated from the serum of either OVA-tolerized mice or control animals [109]. The mice receiving serum from OVA-tolerized mice displayed reduced airway eosinophilia as well as lower serum levels of total or OVA-specific IgE compared to mock-pretreated animals [109]. The developing tolerance was associated with a significantly higher number of activated Tregs in both mediastinal and celiac lymph nodes. In addition, other studies comparing the influence of pretreatment with serum and serum-isolated exosomes showed that the tolerogenic effect of the serum was mediated by exosomes [109]. In an equine study on recurrent airway obstruction, a severe asthma-like disease similar to human asthma [110], another group identified serum miRNA patterns having potential as biomarkers of severe asthma [111]. Eleven miRNAs were found to be differentially expressed between the case and control horses. Subsequent bioinformatic analysis using experimentally validated target genes of the human homologous miRNAs demonstrated significant enrichment in the pathways regulating EMT and modulating maturation and function of CD4^+^ T cells. Moreover, the bioinformatic patterns were in line with a Th2/Th17 type immune response present in severe equine “asthma” [111]. Compared to healthy controls, the expression of miRNA-125b in serum-EVs was higher in asthmatics and correlated with the severity of the disease [112]. Additionally, serum exosomes miRNA-126 levels were higher in allergic asthmatics than in healthy controls [113]. Quite comparably, lung tissue miRNA-126 levels in mice subjected to an OVA-based AAI model were also higher than those in control animals [113]. Another study started with the identification of a set of miRNAs demonstrating differences in expression between eosinophils obtained from asthmatics and those isolated from healthy individuals [114]. Some of these miRNAs demonstrated differences also when analyzed in serum, such as miR-185-5p discriminating asthmatic from healthy subjects. Moreover, in combination with two other miRNAs, miR-185-5p made it possible to create models even better discriminating both conditions and capable of allocating the asthmatic patients into those with intermittent, mild persistent, moderate persistent, and severe persistent disease [114]. Interestingly, serum levels of miR-185-5p and other differentiating miRNAs remained stable over time in asthmatics, in whom no changes in therapy and clinical parameters or health status occurred [115]. Another human study revealed the potential of miR-122-5p in EVs isolated from plasma and sputum to differentiate between healthy controls and (severe) asthmatics. Besides, considering that miR-122-5p correlated with blood immune cell types and that this miRNA was predicted in the pathway analysis to contribute to the differentiation and the function of lymphocytes, it was speculated that miR-122-5p can drive sub-differentiation of various asthma forms, including neutrophilic from eosinophilic asthma [116]. Finally, some circulating miRNAs also demonstrated the potential to discriminate between healthy people and those with asthma or COPD (miR-146a-5p) [117] or between asthma, asthma-COPD overlap, and COPD (miR-320a) [118].

Selected human studies on asthma demonstrating the biomarker potential of EVs are summarized in Table 3.

## 7. Extracellular Vesicles and Asthma: Microorganisms and Other External Influences

### 7.1. Viruses

Viral and bacterial infections of the respiratory tract are known to induce the release of EVs [119,120,121]. Human rhinoviruses (HRV) are responsible for a significant proportion of the exacerbations of chronic inflammatory diseases of the respiratory tract, such as asthma or COPD. It has also been hypothesized that early childhood HRV infection, especially in combination with an atopic pre-disposition, can lead to the establishment of the allergic asthma phenotype, which can persist into adulthood [120,122,123]. Interestingly, HRV infection in young children has been found to be associated with alterations in the airway secretory miRNAome, including a highly specific additional presence of miR-155 in the EVs of nasal secretions [124]. Since miR-155 plays an important role in EV-mediated immune regulation and fine-tuning of type-1/type-2 homeostasis, changes in respiratory tract expression may represent a mechanism by which HRV infection contributes to asthma pathology [124]. In another study, experimental infection with HRV type 16 could induce procoagulatory changes in the respiratory tract of asthmatics, mediated by increased activity of TF-exposing EVs. In addition, these EV-related pro-coagulant alterations were shown to be dependent on HRV load and associated with both neutrophilic and eosinophilic inflammation [125].

### 7.2. Bacteria

Like eukaryotic cells, gram-negative and gram-positive bacteria also release vesicles, referred to as outer membrane vesicles (OMVs) and bacterial membrane vesicles (BMVs), respectively [126,127]. Repeated intranasal application of indoor dust obtained from bed mattresses or EVs isolated from that dust led in mice to the development of neutrophilic pulmonary inflammation accompanied by lung infiltration of both Th1 and Th17 cells, the changes mimicking a typical picture of neutrophilic asthma or COPD in human [128]. In addition, serum levels of IgG1 against dust were found in atopic asthmatic children to be significantly higher than in atopic but otherwise healthy children or those with rhinitis or dermatitis [128]. Subsequent studies conducted in a larger group of individuals suggested that the presence of IgG antibodies against indoor dust EVs in serum is a major risk factor for the development of asthma, COPD, and lung cancer [129]. On the other hand, intranasal pretreatment with exosomes isolated from *Pseudomonas aeruginosa* was able to ameliorate AHR, allergic inflammation, and atopic sensitization levels in mice subjected to OVA- and HDM-based models of AAI [130]. Furthermore, these protective effects were accompanied by an enhancement of Treg responses and a simultaneous decrease in type-2-driven immune mechanisms [130].

In another study, bacterial EVs in the urine of healthy children as well as children with chronic rhinitis, allergic rhinitis, or atopic asthma were analyzed. The distinct bacterial composition of EVs in urine observed between the groups of the study participants suggested that urinary EVs could be an indicator for the assessment of allergic airway diseases in children [131].

### 7.3. Diet

Mouse macrophages treated with purified bovine milk-derived EVs were found to express classically-activated M1 phenotype rather than an alternatively-activated M2 phenotype [132], the latter known to promote type-2 responses and to be associated with allergic asthma [133]. M1 shift was also observed for peritoneal macrophages obtained from mice first fed with EV-enriched diet and then subjected to intranasal installation of agricultural dust extract, while M2 polarization was seen in animals receiving a normal diet and then exposed to organic dust extract [132].

EVs such as exosomes, carrying miRNA and other types of RNA as well as cytosolic and membrane-bound proteins, are also present in human breast milk [134,135,136,137]. Breast milk EVs possess immunomodulatory features and, together with human milk oligosaccharides, seem to play a crucial role in the development of the infant microbiome, which is very important for the appropriate maturation of the immune system [134,135,138,139]. Interestingly, it has been demonstrated that exosomes in human breast milk show phenotypic differences depending on the maternal sensitization status and lifestyle, which might have consequences for the development of allergy in the offspring [140].

### 7.4. Smoking

Differences in miRNA profiles were observed between EVs isolated from BALF of smokers and non-smokers [141]. Furthermore, treatment with smoker and non-smoker BALF EVs exerted differential effects on the function of human bronchial epithelial BEAS-2B cells, including higher IL-6 secretion when smoker EVs were applied [141].

## 8. Conclusions and Perspectives

The studies presented in this review, whatever their nature, i.e., in vitro, in vivo on animals, in human studies, etc., clearly demonstrate the existence of EV communication between cells known as the crucial players in asthma pathology and, moreover, they also strongly suggest an importance of EV-mediated communication mechanisms for the pathobiology of the disease. This includes the mediation of etiopathogenic effects of environmental factors, e.g., microbes or pollutants, and the role of EVs from external origins, e.g., those present in cow’s milk or secreted by certain bacteria. Some of the studies already characterized components of the cargo of EVs and identified molecules responsible for asthma-related effects, mainly small RNAs, proteins, and lipid mediators. Based on this continuously expanding knowledge, the high diagnostic potential of EVs has been highlighted in a variety of studies. It has been shown that EV-based asthma diagnostics effectively targeting miRNA, proteins, or lipids could be performed in different types of biomaterials such as BALF, sputum, NLF, or serum. Considering the access to biomaterials and the methodological rationale, the analytical approach based on the analysis of serum miRNAs seems particularly promising, irrespective of whether and how the miRNAome patterns in blood and lung correspond to each other. These approaches will certainly be expanded in the future to more precisely identify asthma phenotypes particularly by means of non- or minimally-invasive diagnostic sampling techniques [142]. Finally, several in vitro and animal studies reviewed in this article already show that EVs, not only, but especially those secreted by MSCs, can exert beneficial immunomodulatory effects with anti-asthmatic capacity, suggesting a promising potential for EV-based therapeutic approaches. Although procedures regarding targeting specific cell types and the level of EV (cargo) degradation still need to be further optimized, it seems that modified or designed EVs with a higher propensity to fuse with (endosomal) membranes will offer even better therapeutic abilities [38,39]. As also briefly outlined here, another approach being developed as a possible anti-asthmatic therapeutic strategy may involve the use of inhibitors of EV production, which have been shown to exhibit anti-AAI effects in some studies. However, further basic and clinical studies are needed, which undoubtedly will lead to diagnostic and therapeutic innovations based on their results.

## Figures and Tables

**Table 1:** Basic characteristics of extracellular vesicles of different types [26,27,31,32,33].

	Exosomes	Microvesicles	Apoptotic Bodies
Alternative nomenclature	-	Microparticles, ectosomes	-
Size	10–150 nm	100–1000 nm	800–5000 nm
Origin	Intraluminal vesicles within multivesicular bodies	Plasma membrane and cellular content	Plasma membrane, fragmented cell
Formation mechanism	Fusion of multivesicular bodies with plasma membrane	Outward blebbing of plasma membrane	Shrinkage and programmed death of the cell
Release	Constitutive and/or cell activation	Constitutive and/or cell activation	Apoptosis
Time of release	≥10 min	<1 s	-
Composition	Protein, lipids, coding RNA, noncoding RNA, DNA	Protein, lipids, cell organelles, coding RNA, noncoding RNA, DNA	Cell organelles, proteins, nuclear fractions, coding RNA, noncoding RNA, DNA
Enriched protein markers	CD81, CD63, Alix, Tsg101	Selectins, integrin, CD40	Caspase 3, histones

**Table 2:** Major asthma-related cellular and systemic effects of extracellular vesicles (EVs) released by mesenchymal stem cells (MSCs).

EVs	Source Cells/Tissue	Recipient	Main Effect(-s)	Publication
MVs	Equine amniotic MSC	Horse	Reduction in TNF-α secretion and, to a lesser degree, TGF-β and IL-6 from primary alveolar macrophages	[89]
Exosomes	Human BM-derived MSCs	Human	Upregulation of IL-10 and TGF-β1 secretion from PBMCs of asthmatics and promotion of proliferation and immunosuppressive capacity of Tregs	[90]
EVs	Human/mouse BM-derived MSCs	Mouse	Amelioration of *Aspergillus* extract-induced AAI in sensitized animals	[91]
miR-1470-containing exosomes	Human MSCs	Human	Promotion of Tregs differentiation from CD4^+^ T cells isolated from PBMCs of acute asthmatics	[92]
Exosomes	Mouse adipose tissue-derived MSCs	Mouse	Effective suppression of the maturation of BM-derived DCs as reflected by decreased IL-6 release but augmented IL-10 and TGF-β secretion	[93]
EVs	Human adipose tissue-derived MSCs	Mouse	Reduced symptoms and cellular and molecular features of OVA-induced AAI as well as lung TGF-β levels in OVA-sensitized animals	[94]
Exosomes	Mouse adipose tissue-derived MSCs	Mouse	Attenuating effect on airway remodeling in a model of OVA-induced AAI could be further augmented by genetic modifications of MSCs	[95]

MVs, microvesicles; BM, bone marrow; IL-10, interleukin-10; TGF-β1, transforming growth factor beta 1; PBMCs, peripheral blood mononuclear cells; Tregs, regulatory T cells; AAI, allergic airway inflammation; DCs, dendritic cells; OVA, ovalbumin.

**Table 3:** Selected human studies on asthma demonstrating biomarker potential of extracellular vesicle (EV) analysis.

Analyte	Biological Material	Study Subjects	Major result	Publication
Exosomal miRNAs	BALF	Patients with unprovoked, mild, stable asthma and healthy subjects	A subset of miRNAs allowed a robust separation between patients and controls	[98]
Exosomal proteins	BALF	Patients with asthma, cystic fibrosis, or primary ciliary dyskinesia	A subset of proteins allowed accurate separation of the three diseases	[102]
EV lipids	BALF	Asthmatic subjects and healthy controls exposed or not to second-hand smoke	Levels of several lipids different between asthmatics and control groups	[103]
EV RNA, EV proteins	Inducedsputum	Mild allergic asthmatics both before and after allergen challenge	Presence of diverse RNA, especially short RNA species, and immune-related proteins in the samples	[105]
Exosomal proteins	NLF	Asthmatics, asthmatics with chronic rhinosinusitis, and healthy individuals	Levels of several proteins different between patients with respiratory diseases and controls	[106]
Exosomal miRNA	Serum	Untreated asthmatics with various grades of disease severity and healthy controls	Levels of miRNA-125b higher in patients and correlating with disease severity	[112]
Exosomal miRNA	Serum	Allergic asthma patients and healthy controls	Levels of miRNA-126 higher in asthmatics	[113]
(Exosomal) miRNAs	Serum	Asthmatics and healthy individuals	A set of miRNAs capable of discriminating between asthmatics and controls and ranking disease severity; no changes in miRNA levels over time in patients with stable disease	[100,114]
EV miRNA	Plasma, sputum	Mild-to-moderate or severe asthmatics and healthy controls	Levels of miR-122-5p higher in patients with (severe) asthma	[116]

miRNA, microRNA; BALF, Bronchoalveolar Lavage Fluid; NLF, Nasal Lavage Fluid.
